# A Manual of Psychological Medicine and Allied Nervous Diseases

**Published:** 1884-06

**Authors:** 


**A Manual of Psychological Medicine and Allied Nervous Diseases.** Containing the description, etiology, diagnosis, pathology and treatment of insanity, with especial reference to the clinical features of mental diseases and the allied neurosis, and its medico-legal aspects, with a carefully prepared digest of the lunacy laws in the various States relating to the care, custody and responsibility of the insane. Designed for the general practitioner of medicine. By Edward C. Mann, M. D., member of the New York Medico-Legal Society, etc. With photo-type plates and other illustrations. Philadelphia: P. Blakiston, Son & Co., 1012 Walnut Street. 1883.

Psychological medicine is rapidly assuming the proportions of a well-defined and harmonious system of science. It has advanced from small beginnings and unsettled proportions, till today it has become the adjunct of jurisprudence in settling a class of difficult and otherwise inexplicable questions which arise in the administration of justice. The appearance of Dr. Mann's excellent treatise is timely, for it is of importance that the general practitioner should have the means of conveniently informing himself in the latest advances and the outcome of the practical experience of the distinguished men who are laboring in this important field. The description, etiology, diagnosis, pathology and treatment of insanity, and also its medico-legal aspects are intelligently treated, and the value of the book is further increased by a carefully prepared digest of the lunacy laws in the various States relating to the care, custody and responsibility of the insane. We can heartily commend the work to our readers as one worth buying.

